# Collagen type IV alpha 1 (COL4A1) and collagen type XIII alpha 1 (COL13A1) produced in cancer cells promote tumor budding at the invasion front in human urothelial carcinoma of the bladder

**DOI:** 10.18632/oncotarget.16432

**Published:** 2017-03-21

**Authors:** Makito Miyake, Shunta Hori, Yosuke Morizawa, Yoshihiro Tatsumi, Michihiro Toritsuka, Sayuri Ohnishi, Keiji Shimada, Hideki Furuya, Vedbar S. Khadka, Youping Deng, Kenta Ohnishi, Kota Iida, Daisuke Gotoh, Yasushi Nakai, Takeshi Inoue, Satoshi Anai, Kazumasa Torimoto, Katsuya Aoki, Nobumichi Tanaka, Noboru Konishi, Kiyohide Fujimoto

**Affiliations:** ^1^ Department of Urology, Nara Medical University, Kashihara-shi, Nara 634-8522, Japan; ^2^ Department of Pathology, Nara Medical University, Kashihara-shi, Nara 634-8522, Japan; ^3^ Department of Psychiatry, Nara Medical University, Kashihara-shi, Nara 634-8522, Japan; ^4^ Department of Pathology, Nara City Hospital, Nara-shi, Nara 630-8305, Japan; ^5^ Clinical and Translational Research Program, University of Hawaii Cancer Center, Honolulu, HI 96813, USA; ^6^ Bioinformatics Core, Department of Complementary and Integrative Medicine, University of Hawaii John A. Burns School of Medicine, Honolulu, HI 96813, USA

**Keywords:** bladder cancer, tumor budding, invasion, collagen

## Abstract

Current knowledge of the molecular mechanism driving tumor budding is limited. Here, we focused on elucidating the detailed mechanism underlying tumor budding in urothelial cancer of the bladder. Invasive urothelial cancer was pathologically classified into three groups as follows: nodular, trabecular, and infiltrative (tumor budding). Pathohistological analysis of the orthotopic tumor model revealed that human urothelial cancer cell lines MGH-U3, UM-UC-14, and UM-UC-3 displayed typical nodular, trabecular, and infiltrative patterns, respectively. Based on the results of comprehensive gene expression analysis using microarray (25 K Human Oligo chip), we identified two collagens, *COL4A1* and *COL13A1*, which may contribute to the formation of the infiltrative pattern. Visualization of protein interaction networks revealed that proteins associated with connective tissue disorders, epithelial-mesenchymal transition, growth hormone, and estrogen were pivotal factors in tumor cells. To evaluate the invasion pattern of tumor cells *in vitro*, 3-D collective cell invasion assay using Matrigel was performed. Invadopodial formation was evaluated using Gelatin Invadopodia Assay. Knockdown of collagens with siRNA led to dramatic changes in invasion patterns and a decrease in invasion capability through decreased invadopodia. The *in vivo* orthotopic experimental model of bladder tumors showed that intravesical treatment with siRNA targeting *COL4A1* and *COL13A1* inhibited the formation of the infiltrative pattern. COL4A1 and COL13A1 production by cancer cells plays a pivotal role in tumor invasion through the induction of tumor budding. Blocking of these collagens may be an attractive therapeutic approach for treatment of human urothelial cancer of the bladder.

## INTRODUCTION

Urothelial cancer of the bladder (UCB) is the second most frequent neoplasm of the urogenital tract [1]. UCB is known to be a heterogeneous disease. Non-invasive, well-differentiated tumors (Ta) are relatively indolent, but T1 high-grade-UCB and muscle invasive bladder cancer (≥ T2; MIBC) are known to be life-threatening [2]. Despite advancements in multidisciplinary approaches to treatment, management of these diseases remains challenging and controversial. To improve outcomes, the mechanisms underlying tumor invasion, metastasis, and treatment resistance need to be clarified.

Tumor tissue is composed of cancer cells and various types of stromal cells including endothelial cells, macrophages, and fibroblasts. Their interaction and crosstalk might lead to the formation of a cancer-specific microenvironment for tumor invasion and metastasis. Tumor budding is a pathological condition at the tumor invasion front in which individual tumor cells and/or small clusters of tumor cells invade the stromal area [3, 4]. Tumor budding has been reported to be associated with aggressive phenotypes and poor clinical outcomes in various cancers such as colorectal, esophageal, pancreatic, lung, and breast cancers [3–8]. A recent report by Fukumoto *et al*. demonstrated that tumor budding was significantly associated with lymphovascular invasion and poor progression-free survival rate [9]. The pathological evaluation of growth patterns at the tumor invasion front in human UCB was first reported by Jimenez et al. in 2000 [10]. The authors classified human invasive UCB into three groups as follows: nodular, trabecular, and infiltrative. The concepts and definitions of infiltrative growth and tumor budding are similar. Subsequent studies including our reports demonstrated that this pathological parameter was a significant prognostic factor for both T1 tumors and MIBC [2, 11–15].

Tumor budding is characterized by disorganization at the tumor architecture level and dedifferentiation at the cellular level. This phenotype is a dynamic process with loss of cell-to-cell junctions, loss of cell-to-basal membrane, loss of cell polarity, versatile cell shape, and presence of cytoplasmic microfilaments and pseudopodia [3, 16]. The expression and membrane localization of E-cadherin and other adhesion molecules are consistently reduced in tumor budding of malignancies including colorectal cancer [3, 16]. Loss of membranous expression of E-cadherin is one of the hallmarks of epithelial-mesenchymal transition (EMT). Previous studies demonstrated that EMT-inducing signaling pathways including TGF-β and Wnt/β-catenin were activated in tumor budding [17]. To date, little has been reported on the connection between EMT and tumor budding, *i.e*. infiltrative growth pattern, in UCB. In particular, potential molecular events that can trigger tumor budding remain unclear in this malignancy.

A complete understanding of the molecular mechanisms driving tumor budding is essential for overcoming treatment resistance and improving patient outcomes. Based on accumulating evidence of the clinical relevance of tumor budding and infiltrative growth patterns, we investigated the detailed molecular mechanism underlying tumor growth pattern in the present study.

## RESULTS

### Different growth patterns in orthotopic bladder tumors in mice

**Figure 1:**
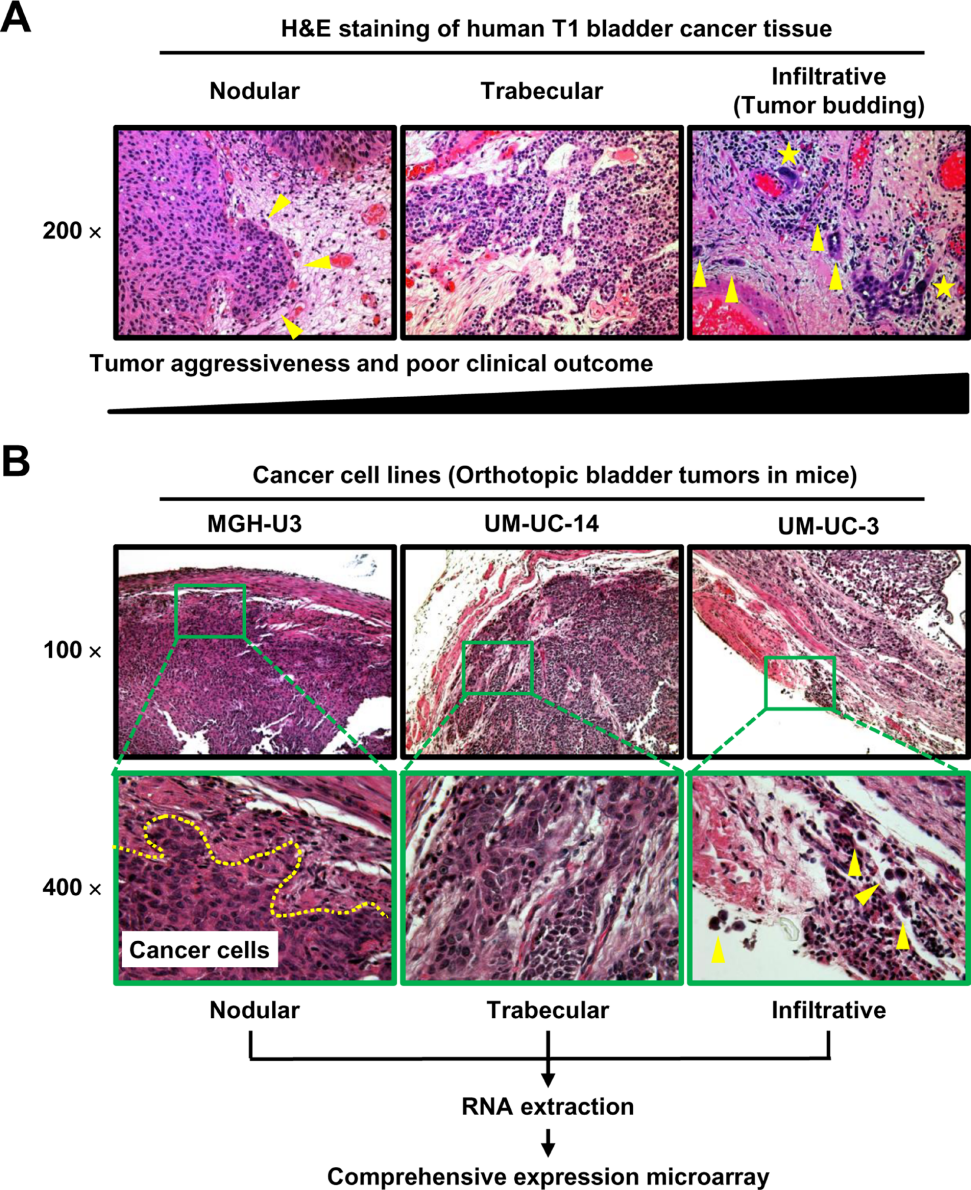
Representative images of H&E-stained specimens of three tumor growth patterns in human UCB (A) and bladder tumors in an orthotopic animal model (B) (**A**) Nodular (left), trabecular (middle), and infiltrative (right) tumor growth patterns shown in the stroma around the tumor area. Yellow arrowheads indicate isolated tumor cells or small clusters of tumor cells (buds). Yellow stars indicate tumor cells with high N/C ratio and abnormal shape. According to previous reports [2, 11–15], the nodular pattern is the most indolent phenotype, while the infiltrative growth pattern (tumor budding) is the most aggressive phenotype and is associated with poor outcomes. (**B**) All images were captured at 100× and 400× magnification. The dashed line in the 400× image of an MGH-U3 tumor indicates the invasion front. Yellow arrowheads in the 400× image of a UM-UC-3 tumor indicate cells that invade the bladder stroma as single cells, with some cells even penetrating the bladder wall. One visible bladder tumor from each cell line was subjected to RNA extraction and cDNA microarray analysis.

Representative H&E images of human UCB tissue show the three patterns; nodlular, trabecular, ad infiltrative patterns (Figure 1A). Four urothelial cancer cell lines were inoculated into the bladder cavities of C.B- 17/severe combined immunodeficiency (SCID) mice. H&E staining of the resected bladders revealed that all cell lines except J82 consistently developed solid bladder masses at 4 weeks after inoculation. In the detailed examination of the murine bladder wall, MGH-U3, UM-UC-14, and UM-UC-3 displayed typical nodular, trabecular, and infiltrative growth patterns, respectively (Figure 1B). UM-UC-3 cells invaded the bladder stroma as single cells, and some cells even penetrated the bladder wall. One visible bladder tumor from each cell line was selected for RNA extraction and cDNA microarray analysis.

### Upregulation of COL4A1 and COL13A1 in aggressive infiltrative growth pattern

**Figure 2:**
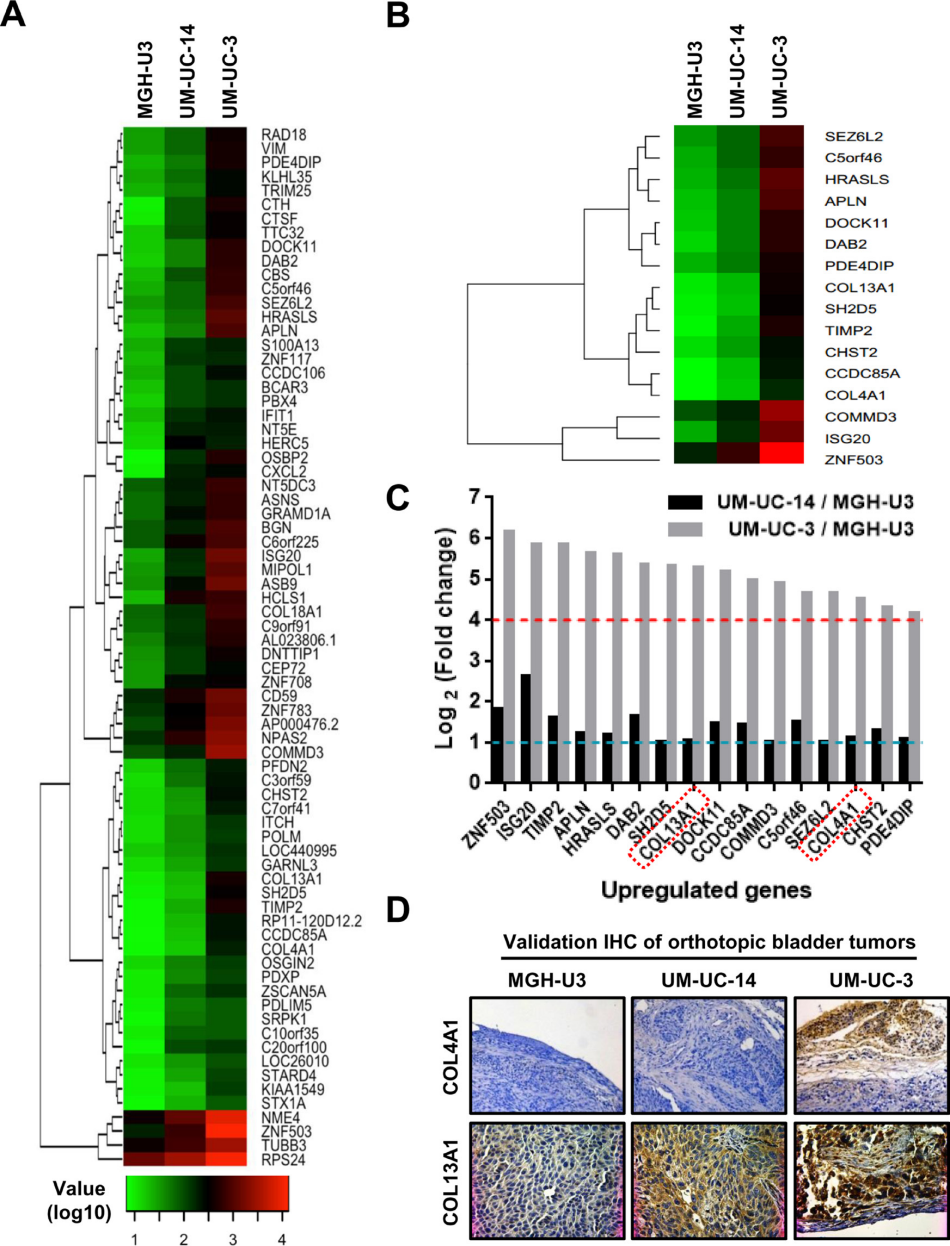
Genome-wide expression analysis in orthotopic bladder tumors resected from SCID mice (**A**) Heatmap representing 74 probes showing > 2-fold upregulation in UM-UC-14 vs. MGH-U3 and > 8-fold upregulation in UM-UC-3 vs. MGH-U3. Heatmap was created by log_10_ transformation of global normalized gene expression values, and probes are ordered based on the complete-linkage hierarchical clustering method. (**B**) Heatmap representing 16 significant probes with > 2-fold upregulation in UM-UC-14 vs. MGH-U3 and > 8-fold upregulation in UM-UC-3 vs. UM-UC-14. Global normalized gene expression values of selected probes were log_10_ transformed, and probes are clustered in rows based on the complete-linkage hierarchical clustering method. (**C**) We applied criteria to identify potentially significant genes with > 2-fold upregulation (log_2_ fold-change > 1, dashed blue line) in UM-UC-14 vs. MGH-U3 and > 8-fold upregulation (log_2_ fold-change > 3, dashed red line) in UM-UC-3 vs. UM-UC-14. Two collagens, COL4A1 and COL13A1, were selected for further analysis (red rectangles). (**D**) Validation of expression levels by immunohistochemical staining. Representative images of three orthotopic bladder tumors resected from SCID mice and stained for COL4A1 and COL13A1. Staining results are consistent with those of microarray profiling. All images were captured at 100× magnification.

To gain insights into mechanisms underlying the formation of the aggressive growth pattern, we performed comprehensive gene expression analysis for MGH-U3 (nodular pattern), UM-UC-14 (trabecular pattern), and UM-UC-3 (infiltrative pattern). Standard selection criteria to identify genes that were significantly upregulated in the infiltrative pattern (lowest expression in MGH-U3, intermediate expression in UM-UC-14, and highest expression in UM-UC-3) were as follows: > 2-fold upregulation (log_2_ fold-change > 1) in UM-UC-14 vs. MGH-U3 and > 8-fold upregulation in UM-UC-3 (log_2_ fold-change > 3) vs. MGH-U3. Out of 24460 genes, 74 met these criteria, and a heatmap was generated based on their expression profiles (Figure 2A). To search for potentially significant genes, we applied the following additional criteria: > 2-fold upregulation (log_2_ fold change > 1) in UM-UC-14 vs. MGH-U3 and > 8-fold upregulation (log_2_ fold change > 3) in UM-UC-3 vs. UM-UC-14. Among 74 genes selected by the initial screening (Figure 2A), 16 genes were identified as strong candidates as shown in Figure 2B and 2C. Among these 16 candidates, some genes, including *ZNF503* and *ISG20*, have been previously found to have oncogenic roles [18, 19]; however, other genes, including *HRASLS* and *DAB2*, have been reported to play a role in tumor suppression [20, 21]. Notably, two collagen genes, collagen type IV A1 (*COL4A1*) and collagen type XIII A1 (*COL13A1*), were included in the list and were upregulated by 23-fold and 38-fold, respectively, in UM-UC-3 tumors as compared to MGH-U3 tumors. We hypothesized that altered expression levels of these collagens may contribute to the formation of infiltrative growth patterns.

Immunohistochemical (IHC) staining of the murine bladders was performed to validate the results of the microarray (Figure 2D). COL4A1 and COL13A1 predominantly localized to the stromal area around the tumor site and the cytoplasm of tumor cells, respectively. There was almost no expression of COL4A1 in MGHU-3 and UM-UC-14 cells, whereas high expression was observed in the stromal area of the UM-UC-3-derived bladder tumor. MGH-U3, UM-UC-14, and UM-UC-3 tumors showed weak, intermediate, and high expression of COL13A1, respectively.

**Figure 3:**
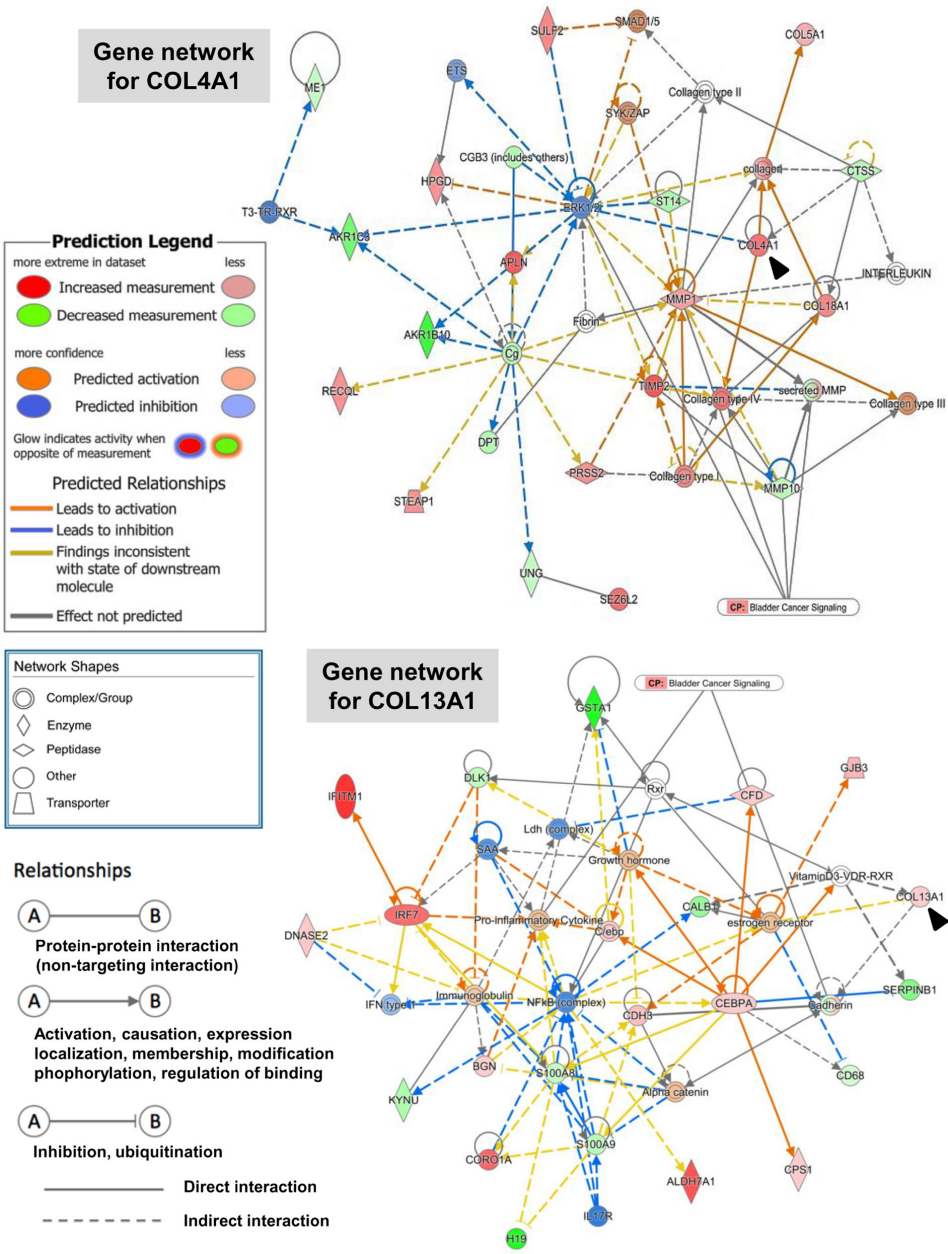
Gene network containing COL4A1 and COL13A1 generated through ingenuity pathway analysis Gene networks for COL4A1 (top) and COL13A1 (bottom) and several of their interaction partners were generated through Ingenuity Pathway Analysis (IPA). Black arrowheads indicate COL4A1 and COL13A1. Colored nodes are shaded by their relative expression (green with low expression and red with higher expression), with color intensity indicating relative expression. The shape of the node indicates the major function of the protein. Lines denote biding of the products of the two genes, while a line with an arrow denotes that one protein acts on another. A dotted line denotes an indirect relationship, and a solid line denotes a direct relationship.

To investigate genes whose expression levels were altered by at least 2-fold in UM-UC-14 vs. MGH-U3, in UM-UC-3 vs. MGH-U3, and in UM-UC-3 vs. UM-UC-14, these genes and their corresponding log_2_ fold-change values were analyzed with IPA software. The core analysis function in IPA revealed 22 biological networks from our dataset containing 279 differentially expressed genes (161 up- and 118 down-regulated) in UM-UC-3 vs. MGH-U3. From among these 22 networks, we identified only one network containing COL4A1, which was associated with connective tissue disorders, organismal injury and abnormalities, and cancer (Figure 3, top). The network included invasion-related proteins (MMP1, MMP19, and TIMP2) and other collagens (COL5A1 and COL18A1). COL4A1 and COL13A1 were found in separate networks according to IPA. COL13A1 was found in a network that was associated with dermatological diseases and conditions, organismal injury and abnormalities, and cancer Figure 3, bottom. This network included EMT-related proteins (cadherin and catenin), pro-inflammatory cytokines, growth hormones, and estrogen.

### IHC analysis of COL4A1 and COL13A1 in murine and human bladder tumors

**Figure 4:**
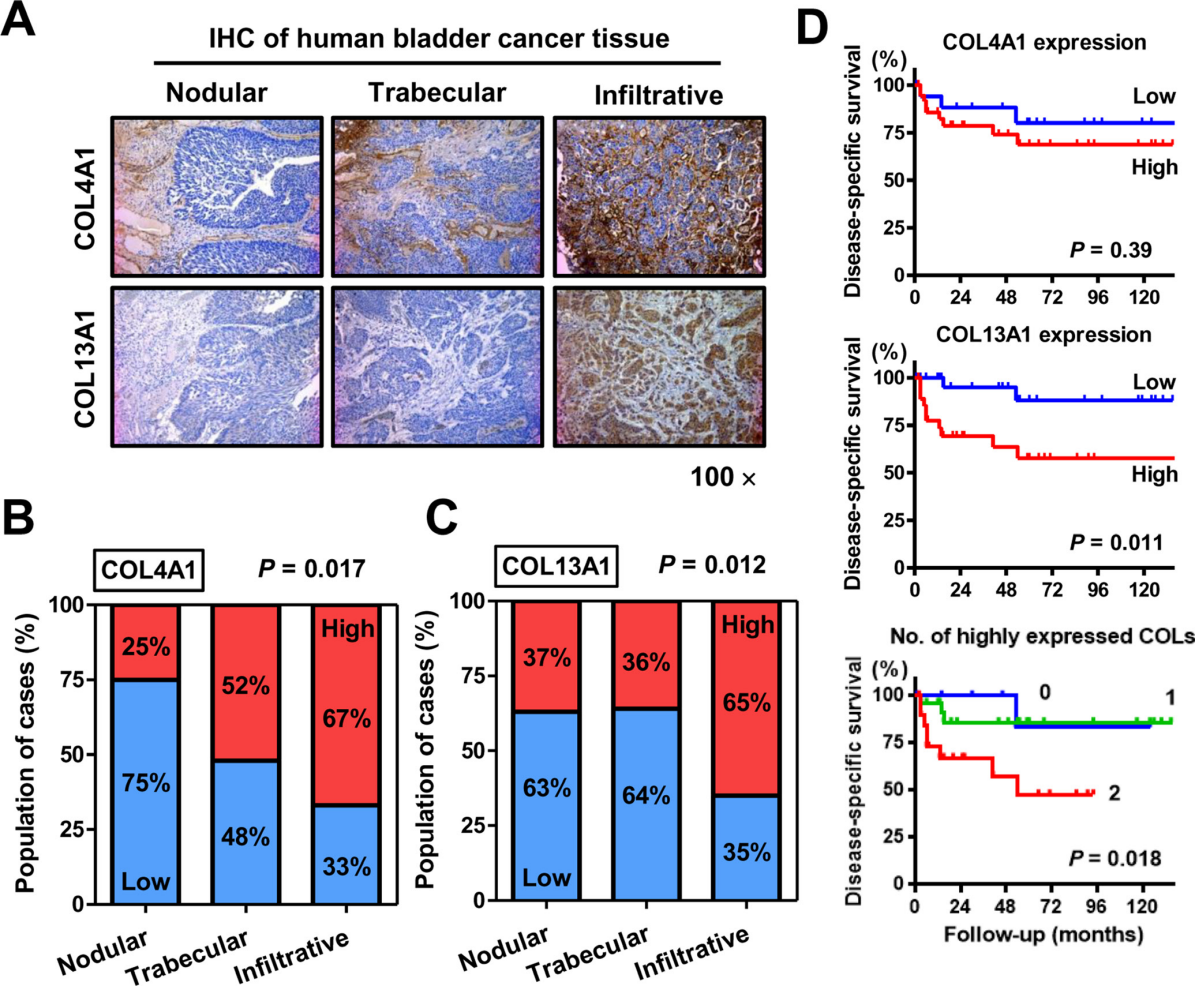
Immunohistochemical (IHC) staining analysis of COL4A1 and COL13A1 in bladder tumor tissues (**A**) Representative images of human UCB with nodular, trabecular, and infiltrative growth patterns. COL4A1 and COL13A1 expression levels are increased in T1 high-grade tumors with infiltrative growth patterns. All images were captured at 100× magnification. (**B**, **C**) Quantification of expression levels of COL4A1 (B) and COL13A1 (C) in three growth patterns. Blue bars and red bars indicate percentages of the population with low expression and high expression, respectively. Data are based on analysis of 97 human bladder tumors. (**D**) Disease-specific survival curves of patients with MIBC after radical cystectomy according to expression levels of COL4A1 (top) or COL13A1 (middle) or according to the number of highly expressed collagens (bottom). The log-rank test (top, middle) or the log-rank test for trend (bottom) was used for comparison.

IHC evaluation of human UCB tissues obtained from 97 patients revealed similar staining patterns for COL4A1 and COL13A1 as those in the orthotopic murine bladder tumors. Figure 4A shows representative images of human UCB tissues with nodular, trabecular, and infiltrative patterns. An example of a tumor with a trabecular pattern shows heterogeneous COL4A1 expression in the stroma around the tumor lesion (Figure 4A, upper-middle panel). The tumor with the infiltrative pattern exhibited strong collagen expression. COL4A1 and COL13A1 expression was notably increased in the trabecular and infiltrative patterns compared with that in the nodular pattern (*P* = 0.017 and 0.012, respectively; Figure 4B and 4C), implying that the expression of these two collagens increases biological aggressiveness and tumor invasiveness in human UCB. Moreover, COL4A1 and COL13A1 expression was associated with higher T category (*P* = 0.045 and 0.041, respectively; Supplementary Table 1).

Of the 97 patients, 57 (59%) were diagnosed with MIBC and treated with RC. Kaplan–Meier analysis of MIBC patients showed that high expression of COL13A1, but not COL4A1, was significantly associated with poor outcomes after RC (Figure 4D). When patients were stratified into three groups depending on the number of highly-expressed collagens, 11 (19%) patients exhibited high expression of neither COL13A1 nor COL4A1, 6%) exhibited high expression of one of these, and the remaining 20 (35%) exhibited high expression of both. The hazard ratios were and 1.28 (95% confidence interval: 0.15–10.9) and 3.51 (1.21–13.6) for cases with one and two highly expressed collagens, respectively, compared with that of cases with neither collagen highly expressed, showing that disease-specific survival (DSS) decreased dramatically as the number of highly expressed collagens increased (Figure 4D).

### Change in invasion pattern and capability following downregulation of COL4A1 and COL13A1

**Figure 5:**
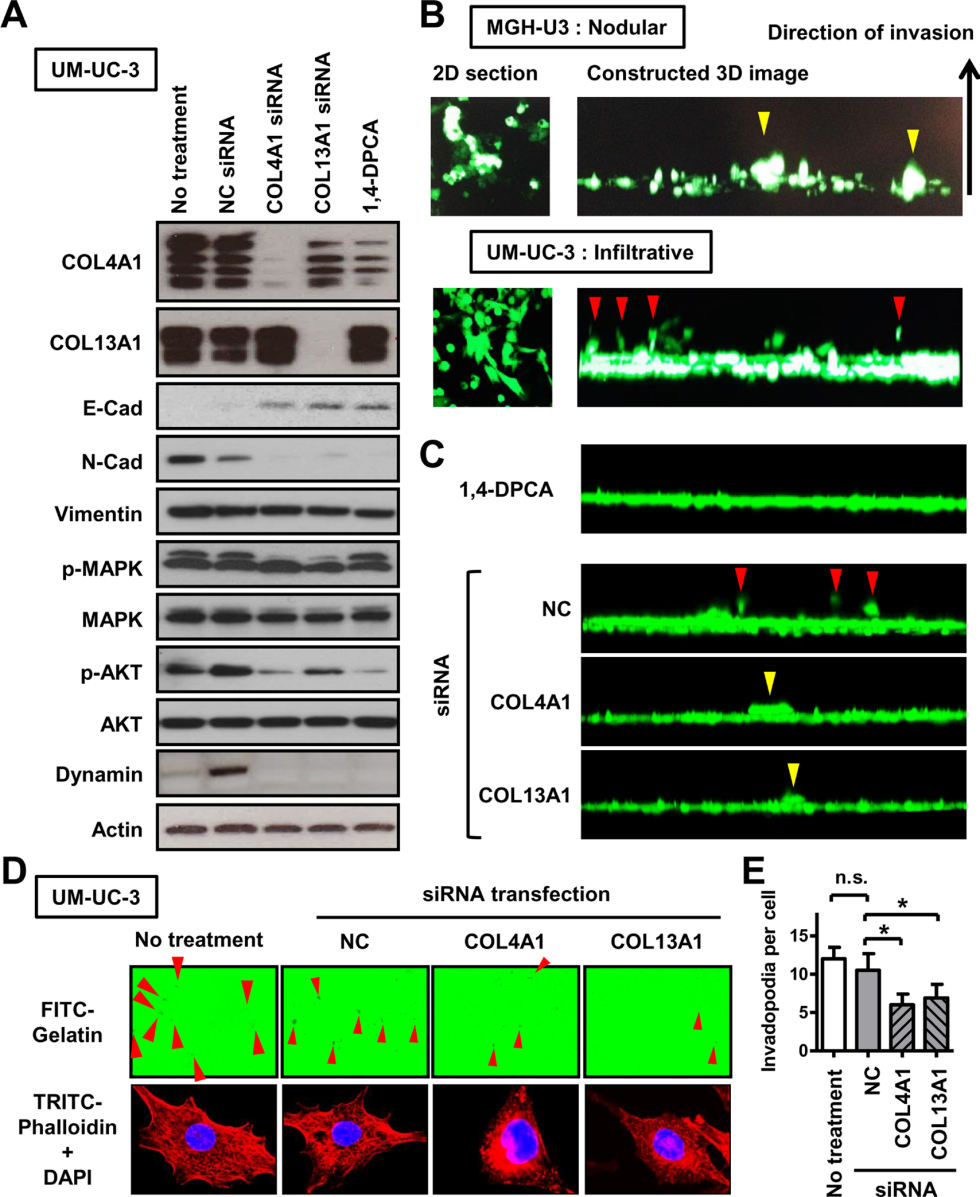
The association of COL4A1 and COL13A1 produced in UCB cells with the formation of the infiltrative growth pattern (**A**) EMT-related markers, proteins involved in the activation of intracellular signaling pathways, and an invadopodia-related marker were quantified by western blot analysis in UM-UC-3 cells, which has high endogenous expression of COL4A1 and COL13A1 proteins. The list of antibodies and conditions are shown in Supplementary Table 3. Actin was used as a loading control. Four bands for COL4A1 and two bands for COL13A1 were detected. (**B**) Representative images of results of 3-D collective inverse invasion assay of MGH-U3 and UM-UC-3 cells. Optical 2-D sections of cells stained with calcein AM invading Matrigel, taken at 10-μm intervals by confocal microscopy. Right panels represent reconstructed 3-D images of cell invasion from a stack of confocal Z-series images, viewed from the side. Yellow and red arrowheads indicate nodular invasion pattern of MGH-U3 and infiltrative invasion pattern of UM-UC-3, respectively. (**C**) Representatives of 3-D images of UM-UC-3 cells treated with 1 μM 1,4-DPCA or transfected with NC siRNA, COL4A1 siRNA, or COL13A1 siRNA are shown. Red and yellow arrowheads indicate infiltrative and nodular invasion patterns, respectively. (**D**) Representative images of FITC-gelatin degradation invadopodia assay using non-treated or NC siRNA-, COL4A1 siRNA-, or COL13A1 siRNA-transfected UM-UC-3 cells. Co-localization of matrix degradation spots with phalloidin-stained F-actin validates these foci as invadopodia. Red arrowheads indicate formed invadopodia. Images were captured by microscope objective lens with synthetic oil immersion at high magnification of 1,000×. (**E**) The number of formed invadopodia was counted and quantified for 20 cells in each group. Data are expressed as the mean ± SD of three independent experiments; *P < 0.05.

To confirm that these two collagens produced in UCB are associated with the formation of the tumor growth pattern, *in vitro* experiments using MGH-U3 and UM-UC-3 were performed with siRNA transfection targeting collagens and 1,4-dihydrophenonthrolin-4-one-3- carboxylic acid (1,4-DPCA) a potent inhibitor of collagen deposition. To determine the 50% inhibitory concentration (IC50) of 1,4-DPCA, proliferation sigmoid curves were generated as previously described [22]. The IC50 values in MGH-U3, UM-UC-14, and UM-UC-3 cells were 4.3, 8.4, and 10.3 μM, respectively (Supplementary Figure 1). In order to avoid affecting proliferation, 1 μM (almost no effect on cell proliferation) of 1,4-DPCA was used for further experiments. Western blot analysis of UM-UC-3 confirmed effective downregulation of COL4A1 and COL13A1 at the protein level by siRNA transfection (Figure 5A). In contrast, 1,4-DPCA inhibited the production of COL4A1, but did not affect the level of COL13A1.

Based on the results of network analysis (Figure 3), we analyzed levels of EMT-related proteins (cadherins and vimentin), an invadopodia-related protein (dynamin), and intracellular signaling pathway proteins (MAPK and AKT) after inhibition of endogenous collagens. On the basis of the loss of E-cadherin and the expression of N-cadherin and vimentin in parental UM-UC-3 cells, this cell line could be characterized as aggressive. E- to N-cadherin switching was observed following downregulation of COL4A1 and COL13A1, indicating an association between the two collagens and the promotion of EMT. In addition, our analysis suggested that both collagens could be activators of the PI3K/AKT pathway, which plays critical roles in regulating diverse cellular functions including metabolism, growth, proliferation, survival, transcription, and protein synthesis. Integrins are transmembrane receptors consisting of one α and one β subunit and are involved in various cellular functions including cell growth, differentiation, would healing, angiogenesis, and vasculogenesis [23]. Integrins α1β1 and α2β1 have been reported to act via collagen binding. We examined if the expression levels of the genes encoding integrins α2 and β1 were altered by knockdown of COL4A1 and COL13A1. However, these expression levels were not affected by knockdown of their ligands (Supplementary Figure 2).

When the growth patterns at the invasion fronts of UCB cells were examined using a 3-D matrix comprised of Matrigel, MGH-U3 cells invaded as well-formed nodular-like collectives, whereas UM-UC-3 cells invaded in spindle-shaped single cells, consistent with the infiltrative pattern (Figure 5B). However, downregulation of collagens dramatically decreased invasion capability and altered invasion patterns (Figure 5C). Individual knockdown of either COL4A1 or COL13A1 hampered the formation of the infiltrative pattern and cell budding and resulted in the nodular pattern, which is the most indolent phenotype. Tumor invasion requires the initiation of path generation in leading cells and proteolytic remodeling of the extracellular matrix (ECM). To investigate whether collagens produced in cancer cells promote invadopodial formation, UM-UC-3 cells were seeded on wells coated with fluorescent gelatin. UM-UC-3 cells without any treatment (control) exhibited many F-actin-rich protrusions into the gelatin, which were indicated by dark spots generated by degradation of the protein matrix (Figure 5D, left). Co-localization of matrix degradation spots with phalloidin-stained F-actin validates these foci as invadopodia. Collagen knockdown reduced the number of invadopodial structures per cell (Figure 5D and 5E) and the expression of dynamin I/II.

Our findings suggest that COL4A1 and COL13A1 produced in UCB cells promote invasive capability and the formation of the infiltrative growth pattern via the upregulation of EMT and invadopodia-related proteins through activation of the AKT pathway.

### Confirmatory *in vivo* study of intravesical collagen blockade therapeutic approach

**Figure 6:**
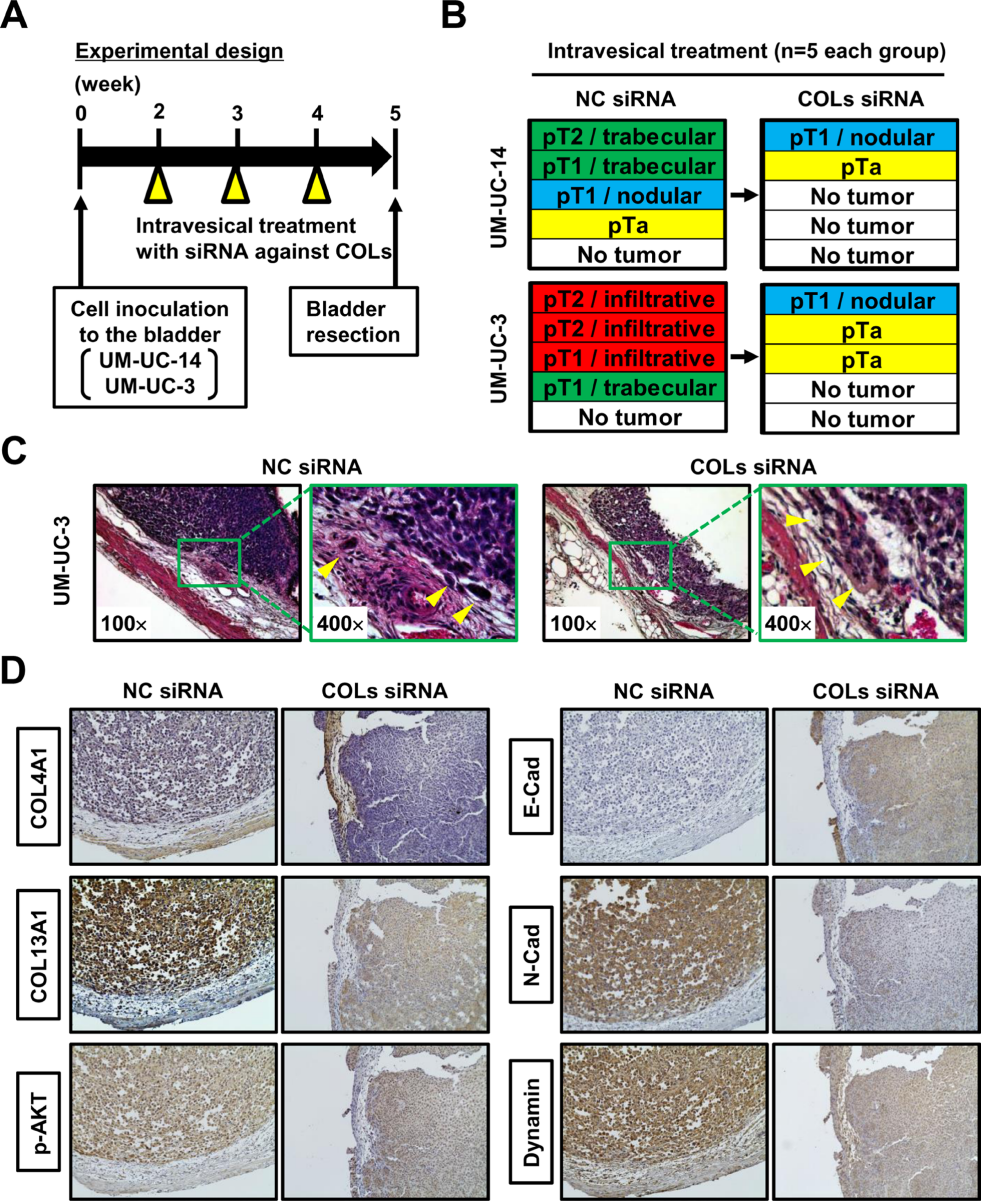
Orthotopic animal experiments for intravesical collagen blockade therapeutic approach (**A**) Experimental design used in this study. (**B**) After completion of treatment, resected bladders from each group (*n* = 5/group) were subjected to H&E staining. Pathological diagnoses of NC siRNA-treated bladder tumors and COLs siRNA-treated bladder tumors were compared in two UCB cell lines. (**C**) Representative H&E images of NC siRNA-treated bladder tumor and COLs siRNA-treated bladder tumors demonstrated inhibition of the formation of the infiltrative growth pattern following COLs siRNA treatment. All images were captured at 100× and 400× magnification. (**D**) Representative image of immunohistochemical staining of NC siRNA-treated bladder tumor and COLs siRNA-treated bladder tumors. The images demonstrate the modulation of proteins related to the expression of COLs. All images were captured at 100× magnification.

To investigate the therapeutic potential of collagen blockade as an intravesical approach, we created an *in vivo* orthotopic experimental model of bladder tumor and performed siRNA-mediated inhibition of collagens. The experimental design is shown in Figure 6A. Two weeks after intravesical implantation of UM-UC-14 and UM-UC-3, intravesical treatment using siRNA against both COL4A1 and COL13A1 (COLs siRNA) or NC siRNA was performed once a week for three weeks (*n* = 5 each treatment group). One week after completion of the intravesical therapy, animals were euthanized and bladders were harvested. We pathologically evaluated the T category and tumor growth pattern when the T category was ≥ pT1. The sizes and weights of resected bladder tumors treated with COLs siRNA were not apparently different from those treated with NC siRNA (data not shown). A comparison of the pathological diagnoses of NC siRNA-treated bladder tumors and COLs siRNA-treated bladder tumors is depicted in Figure 6B. Of five UM-UC-14-derived bladder tumors treated with NC siRNA, two (40%) showed trabecular growth patterns, and one (20%) showed a nodular growth pattern. When treated with COLs siRNA, one mouse (20%) developed a T1 tumor with a nodular growth pattern, one had a small Ta tumor, and the remaining three (60%) did not show any evidence of tumor growth. Of five UM-UC-3-derived bladder tumors treated with NC siRNA, three (40%) showed infiltrative growth patterns, and one (20%) showed a trabecular growth pattern. When treated with COLs siRNA, one mouse (20%) developed a T1 tumor with a nodular growth pattern, two (40%) had Ta tumors, and the remaining two did not show any evidence of tumor growth. Representative images of NC siRNA-treated bladder tumors and COLs siRNA-treated bladder tumors demonstrated inhibition of the formation of the infiltrative growth pattern (Figure 6C). Thus, COL4A1 and COL13A1 production in cancer cells supports their high invasion capability into the bladder wall and promotes subsequent tumor progression and metastasis in orthotopic bladders.

Immunostaining analysis of orthotopic bladder tumors showed that both COL4A1 and COL13A1 were knocked down by intravesical treatment with COLs siRNA (Figure 6D). Along with the knocking down of two collagens, decreased phosphorylated AKT, N-cadherin, and dynamin were observed whereas increased membranous expression of E-cadherin was seen. These results were compatible with those of in-vitro analysis (Figure 5A).

## DISCUSSION

Migration and invasion of cancer cells are heterogeneous processes consisting of cell–cell and cell–matrix adhesion, cytoskeletal polarity, and pericellular proteolysis under pathological conditions [24, 25]. Structural and molecular modulation of cancer cell behavior and the peritumoral environment determine whether cells migrate and invade stromal tissues collectively (nodular/trabecular pattern) or individually (infiltrative pattern) (Figure 1). Friedl *et al*. reviewed detailed mechanisms of cell migration and classified them according to migration modes: collective, amoeboid, or mesenchymal [24]. Each migration mode is controlled minutely and dynamically by a set of molecular mechanisms. For instance, collective cell migration allows epithelia, ducts, glands, and vessels to build and regenerate complex tissues, but it also contributes to cancer progression by local invasion [26]. In contrast, amoeboid/mesenchymal single-cell migration is essential for neural crest cell integration into tissues, for moving to another location in the body, and for fulfilling effector functions for immune cell trafficking [24, 27]. This process is linked strongly with cancer remote metastasis, in which cancer cells migrate and invade individually though the ECM to circulate in the bloodstream [28]. In a recent publication, the infiltrative pattern and tumor budding at the tumor invasion front were significantly associated with high malignant potential and poor clinical outcome in both MIBC and MIBC as well as colorectal cancer [2, 9, 14, 15]. However, the molecular keys to each migration mode are not yet fully understood.

We implemented a unique experimental strategy for generating animal models with nodular, trabecular, and infiltrative growth patterns in the bladder. RNA expression patterns are significantly affected by the environment in which tumor cells grow [29, 30]. A previous study revealed that genes with a pattern of higher expression in *in vivo* xenograft tumors compared with that in *in vitro* tissue culture encoded proteins involved in the ECM and cell surface receptors [30]. In the present study, an orthotopic bladder tumor model in SCID mice was generated on the basis that this model could be expected to show mRNA expression profiles similar to that in human UCB. Mouse bladders were resected and processed for comprehensive gene expression analysis. The analysis of three cell lines with nodular, trabecular, and infiltrative patterns identified several genes that may be associated with the formation of cancer migration and invasion patterns (Figure 2B). Among these candidates, two collagens were selected for further functional analysis. COL4A1 and COL13A1 were up-regulated by 22.6-fold and 38.5-fold, respectively, in UM-UC-3 vs. MGH-U3. COL4A1 and COL13A1 are subunits of the type IV collagen and type XIII collagen, respectively.

Collagen type I is the most abundant collagen in the human body, while collagen type IV is the most abundant and essential in basement membranes (BMs). The BM is a specialized form of ECM consisting of collagen type IV, laminin, fibronectin, and entactin. The BM acts as a critical component regulating tumor cell behavior, as not only a structural feature of tissues but also a functional component of blood vessels, which constitute a microenvironment sensor for endothelial cells and pericytes [31]. A couple of previous reports demonstrated that COL4A1 plays an important role in angiogenesis and tumor progression [31, 32]. Collagen type XIII is a transmembrane protein localized in cell-cell and cell-ECM junctions [33, 34]. This type of collagen has not been well studied, especially in the oncological field. In 2008, Tuomisto *et al*. demonstrated that collagen type XIII is a determinant of BM structure in mouse intestines. This report concluded that collagen type XIII might work normally as a tumor suppressor gene in the development of intestinal microbe-dependent lymphomas [35]. However, there was no investigation into the association between collagen type XIII and disease progression and metastasis.

**Figure 7:**
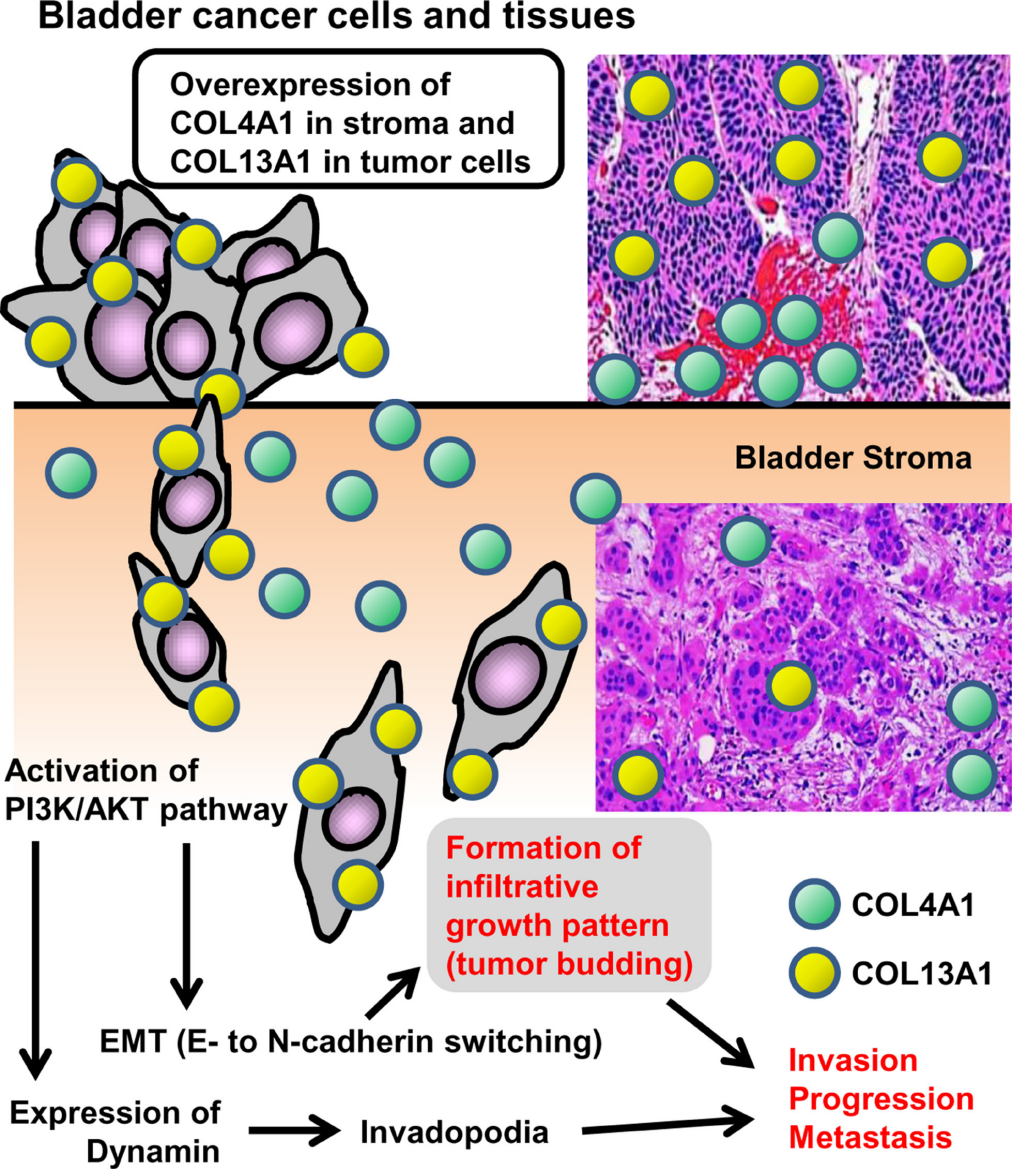
Schematic diagram of the proposed mechanism for COL4A1 and COL13A1 in human urothelial carcinoma of the bladder COL4A1 (green circle) and COL13A1 (red circle) are localized to the stromal area around the tumor site and the cytoplasm of tumor cells, respectively. Overexpression of these two subunits of collagen in the tumor lesion leads to tumor invasion, progression, and eventually metastasis. EMT = epithelial-mesenchymal transition.

The present study investigated the association of COL4A1 and COL13A1 with tumor invasion and progression using *in vitro* and *in vivo* experimental models and clinical materials of human UCB. We first demonstrated that expression of COL13A1 in UCB cells potentially causes a significant increase in invasion capability via the formation of the infiltrative invasion pattern and invadopodia. It has been reported that the activation of TGF-β and Wnt/β-catenin may promote EMT, resulting in the tumor infiltrative pattern or tumor budding [17]. However, our findings demonstrate that COL4A1 and COL13A1 produced in UCB cells activate the intracellular AKT signaling pathway, which leads to an E/N-cadherin switch (Figure 4A). Figure 7 depicts a schematic diagram of the proposed mechanism including the crucial roles of COL4A1 and COL13A1 in human UCB. Previous studies suggested that the induction of collagens including COL1A1 and COL4A1 is regulated by chemokine signaling (CXCR7) and downstream pathways such as PI3K/AKT and NF-κB [31, 32]. COL6A3, an ECM protein, has been identified as the clinically relevant collagen in colorectal, ovarian, and pancreatic cancer [36–38]. COL6A3 protein and mRNA are significantly upregulated not only in colorectal cancer cells but also in the stromal cells of tumor lesions, which promote tumor growth by modulating Hippo and Wnt signaling [36, 39]. The upregulation of COL6A3 protein and mRNA in the cancer stroma predicts poor outcomes in patients with colorectal cancer [36, 39]. Collectively, these data suggest that collagen upregulation in the tumor microenvironment may play a critical role in human cancer.

One limitation of this study is that only one cell line was subjected to microarray analysis for each tumor growth pattern. Usage of multiple cell lines for each pattern would provide more reliable and accurate results. However, we believe that this limitation was not critical because the *in vitro* and *in vivo* experiments for validation successfully revealed the clinical relevance of the selected collagens, COL4A1 and COL13A1.

Our study provides novel and comprehensive insight into the tumor microenvironment of human UCB. Knowledge of the underlying mechanism of the formation of tumor budding and the infiltrative growth pattern will facilitate the search for an alternative treatment approach for targeting the tumor microenvironment of UCB. Novel small-molecule inhibitors, neutralizing antibodies, or anti-sense nucleotides disrupting COL4A1 and COL13A1 should be developed and tested in patients with bladder cancer refractive to conventional therapies.

## MATERIALS AND METHODS

### Patient selection and data collection

The Ethics Committee of the Nara Medical University approved this study, and all participants provided informed consent (reference number: 1256). The study was conducted on 97 patients with pathologically diagnosed UCB who were treated by transurethral resection of bladder tumor (TURBT) and/or radical cystectomy (RC) between 2002 and 2016. Patient demographics are shown in Supplementary Table 2. Clinical information and follow-up data were collected by retrospective chart review. Follow-up was performed according to our institutional protocol [2, 15].

### Assessment of growth patterns and tumor budding in human UCB

The growth pattern at the tumor invasion front and tumor budding were determined histologically by a combination method according to Jimenez's definition [10] and Ueno's definition (10 buds in a 25× field) [40], which is the most commonly applied method in previous studies. Briefly, the nodular pattern was composed of mostly well-formed, rounded, and circumscribed nests of tumor cells invading the stroma. The trabecular pattern was composed of tumor broad trabeculae, which anastomosed with each other. Trabeculae thicknesses are ≥ 3 cells. The infiltrative pattern was composed of isolated single cells or small clusters consisting of a few cells [10]. However, in the present study, we regarded tumors that met the definition of infiltrative pattern defined by Jimenez et al. [10] and those characterized by tumor budding as defined by Ueno et al. [40] as infiltrative tumors. The most aggressive pattern was recorded for each case when tumors displayed more than one pattern (Figure 1A).

### Cell lines, reagents, and siRNA transfection

Four urothelial cancer cell lines, MGH-U3 (a generous gift from Dr. H. LaRue at Laval University Cancer Research Centre, Quebec, Canada), UM-UC-14 (Sigma-Aldrich, St. Louis, MO, USA), J82 (ATCC, Manassas, VA, USA), and UM-UC-3 (ATCC) were used in the present study. Cell lines were authenticated by analysis of their genetic alterations. Cell lines were maintained in RPMI-1640 media supplemented with 10% fetal bovine serum (FBS), 100 units/mL penicillin, and 100 μg/mL streptomycin in a standard humidified incubator at 37°C in 5% CO_2_. An inhibitor of prolyl 4-hydroxylase and collagen deposition, 1,4-DPCA was purchased from Cayman Chemical (Ann Arbor, MI, USA) and dissolved in DMSO.

siRNA transfection was performed by a reverse transfection method as previously described [41]. Double-stranded siRNA targeting human *COL4A1* (siRNA ID# s3287) and *COL13A1* (siRNA ID# s3350), and negative control (NC) siRNA were synthesized by Thermo Fisher Scientific (Waltham, MA, USA). UM-UC-3 cells were transfected for 48 h with siRNA using 6-well plates with 100 pmol of siRNA and 5 μL of Lipofectamine™ RNAiMAX Transfection Reagent (Thermo Fisher Scientific) according to the manufacturer's directions.

### Orthotopic bladder cancer model and treatment design

Approval for the animal studies was obtained from the Committee on Animal Research of the Nara Medical University (reference numbers: 11343 and 11416). Specific pathogen-free 6-week-old female SCID mice were used (Oriental Bio Service, Kyoto, Japan). Four animals were included for each cell line (MGH-U3, UM-UC-14, J82, and UM-UC-3). To generate the orthotopic bladder tumor models, 2 × 10^6^ cells were inoculated into the bladder cavities of SCID mice by 24-gauge angiocatheters (Surflo®; Terumo Corp, Tokyo, Japan) as described previously [41]. Four weeks after inoculation, mouse bladders were resected to evaluate the tumor growth pattern at the invasion front by H&E staining and processed for RNA extraction and comprehensive gene expression analysis.

For analysis of intravesical treatment, UM-UC-14 and UM-UC-3 were implanted into the bladders of female SCID mice. Intravesical treatment of siRNA was performed as previously described [42]. Two weeks later, intravesical treatment was initiated. RNAiMAX reagent (50 μL) containing 100 nM siRNA (cocktail of 50 nM *COL4A1* siRNA and 50 nM *COL13A1* siRNA) was added to 50 μL of PBS to prepare a final volume of 100 μL. The complex was delivered and allowed to dwell in the bladder for 2 h by occlusion of the urethra with a purse string suture. Intravesical therapy was administered weekly for a total of three weeks. One week after completion of intravesical therapy, animals were euthanized, and bladders were harvested and processed for H&E examination.

### Gene microarray and network analysis using ingenuity pathway analysis

Total RNA was extracted from orthotopic bladder tumors of human urothelial cancer cells using Arcturus® Paradise^®^ PLUS 2 Round Kit-Amino Allyl (Life Technologies, Carlsbad, CA, USA) and analyzed with a 3D-Gene Human Oligo chip 25K (Toray Industries, Inc., Tokyo, Japan) according to the manufacturer's protocol (www.3d-gene.com). Obtained RNA was transcriptionally amplified once using the Amino Allyl MessageAMP II aRNA Amplification Kit (Applied Biosystems, CA, USA) and labeled with Cy5 dye (GE Healthcare, Buckinghamshire, England). The Cy5-labeled aRNA pools were mixed with hybridization buffer and hybridized for 16 h. The hybridization signals were obtained using a 3D-Gene Scanner (Toray Industries Inc.) and processed by 3D-Gene Extraction (Toray Industries Inc.). Detected signals for each gene were normalized by a global normalization method (the median of the detected signal intensity was adjusted to 25). A total of 279 genes with fold-changes greater than 2 in all comparisons (UM-UC-14 vs. MGH-U3, UM-UC-3 vs. MGH-U3, and UM-UC-3 vs. UM-UC-14) were used for molecular network and pathway analysis using Ingenuity Pathway Analysis (IPA, http://www.ingenuity.com) software.

### Immunohistochemical staining and quantification

IHC staining using paraffin-embedded, formalin-fixed tissue blocks was performed as previously described [42, 43]. Information on the antibodies used is available in Supplementary Table 3. Only sections with visible stromal or cancer cells were included in the analysis. Quantification of collagen expression was carried out by two investigators (S. Hori and Y. Tatsumi) blinded to knowledge of the patients’ outcomes or other clinicopathological characteristics. Because the dominant localization of COL4A1 was the stromal area around the tumor, immunoreactivity was evaluated semi-quantitatively by the scoring method for cancer-associated fibroblasts as previously described [42], which was based on the number of COL4A1-positive cells in at least three independent fields as follows: 0, none or a small number of positive cells surrounding less than half of the border; 1, a moderate number of positive cells surrounding less than half of the border; or 2, a large number of positive cells surrounding more than half of the border. The expression level of COL4A1 was determined as follows: low (scores 0 or 1) or high (score 2). COL13A1 expression in cancer cells was semi-quantified in at least five independent fields. Briefly, the proportion (0 = 0% of cells; 1 = 1−40%; 2 = 41−75%, and 3 = 76−100%) and intensity scores were added to obtain a combined IHC score [43] ranging from 0 to 6 as follows: low (0–3) or high (4–6).

### Western blot analysis

Western blotting was performed as previously described [42]. Primary antibodies are listed in Supplementary Table 3.

### Three-dimensional (3-D) cell invasion assay

To evaluate the infiltration patterns of tumor cells *in-vitro*, 3-D collective cell invasion assay was performed using phenol red-free growth factor-reduced Matrigel (BD Biosciences, San Jose, CA) and the BD Falcon Insert System as previously reported [44]. The video component of the report can be found at http://www.jove.com/video/3525/ Briefly, 100 μL of Matrigel diluted with PBS (1:1) was pipetted into Transwell inserts with 8-μm pore membranes in a 24-well plate and incubated for 30 min at 37°C to solidify. The Transwell inserts were inverted and 100 μL of the cell suspension (4 × 10^4^ cells of MGH-U3 or UM-UC-3 cells) were pipetted onto the upward facing underside of the membranes. The inserts were covered with the base of a culture plate, making contact with each droplet of cell suspension. After incubation in the inverted state for 4 h to allow cell attachment, the Transwells were turned right side up and placed in wells of a 24-well plate containing serum-free RPMI. Growth medium containing 10% FBS as a chemoattractant was added to the Transwell on top of the solidified Matrigel, and the plate was incubated for 5 days at 37°C. To image tumor cells invading Matrigel, 500 μL of 4 μM calcein AM fluorscent dye (PromoKine, Heidelberg, Germany) was poured on top of each Matrigel plug, followed by incubation for 1 h at 37°C in 5% CO_2_ humidified atmosphere. The Transwells were placed on large coverslips over a non-immersion 20× objective, and optical sections were captured every 10 μm from the bottom of the Matrigel plug using a confocal laser microscope FV1200 (Olympus, Tokyo, Japan). Individual optical slices were used to build 3-D objects using FLUOVIEW software (Olympus).

### Gelatin degradation assay

Invadopodial formation was evaluated using QCM^TM^ Gelatin Invadopodia Assay (Millipore, Billerica, MA, USA; cat No. ECM670) according to the manufacturer's instructions. Briefly, UM-UC-3 was transfected by NC siRNA, *COL4A1* siRNA, or *COL13A1* siRNA. After a 48-h incubation, cells were trypsinized, and 20,000 cells were seeded per well of a Lab-Tek 8-well chamber slide, which was pre-coated with FITC-labeled gelatin. After a 24-h incubation at 37°C, cells were fixed with 4% paraformaldehyde and stained with TRITC-phalloidin and DAPI for immunofluorescence microscopy (Leica DMI 4000B, Wetzlar, Germany). Invadopodia were visible as dark spots devoid of fluorescence. The number of formed invadopodia was counted and quantified for 20 cells in each group.

### Statistical analysis

DSS from the day of RC were obtained using the Kaplan–Meier method and compared by the log-rank test or log-rank test for trend. Data were visualized by bar charts or box plots, and the Student's *t*-test or Mann–Whitney *U*-test was applied for statistical analysis as appropriate. PRISM software version 5.00 (San Diego, CA, USA) was used for statistical analyses and plotting the data. *P* < 0.05 was considered statistically significant.

## SUPPLEMENTARY MATERIALS FIGURES AND TABLES



## Abbreviations

UCB: urothelial cancer of the bladder
MIBC: muscle invasive bladder cancer
EMT: epithelial-mesenchymal transition
DSS: disease-specific survival
1,4-DPCA: 1,4-dihydrophenonthrolin-4-one-3- carboxylic acid
TURBT: transurethral resection of bladder tumor
RC: radical cystectomy
COL4A1: collagen type IV A1
COL13A1: collagen type XIII A1
SCID: severe combined immunodeficiency
IHC: immunohistochemistry
BM: basement membrane
