# Supplementary material for: Collagen type IV alpha 1 (COL4A1) and collagen type XIII alpha 1 (COL13A1) produced in cancer cells promote tumor budding at the invasion front in human urothelial carcinoma of the bladder
Source: Oncotarget. 2017 Mar 21;8(22):36099–114. doi: 10.18632/oncotarget.16432 (PMC5482641; doi:10.18632/oncotarget.16432)
Supplement: Supplementary file 1 [file oncotarget-08-36099-s001.pdf]

Collagen type IV alpha 1 (COL4A1) and collagen type XIII alpha 1 (COL13A1) produced in cancer cells promote tumor budding at the invasion front in human urothelial carcinoma of the bladder

Supplementary Materials

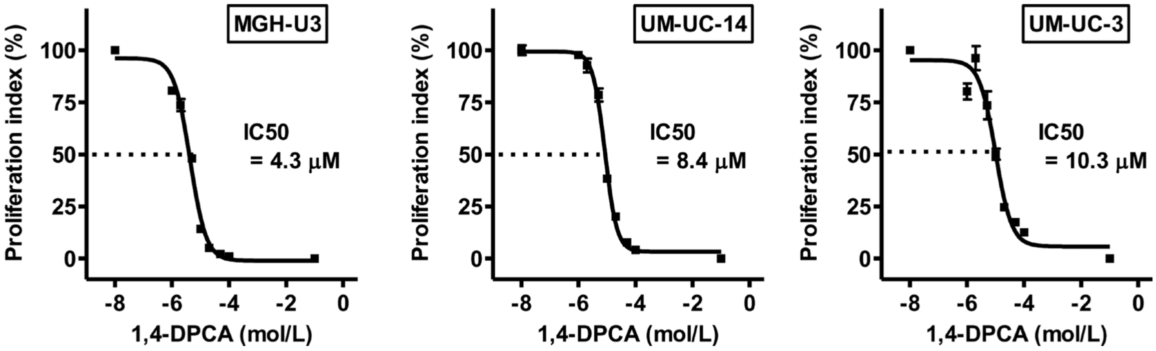

Supplementary Figure 1: Growth inhibitory sigmoid curve analysis of MGH-U3, UM-UC-14, and UM-UC-3 following treatment with 1,4-DPCA. To determine sensitivity against 1,4-DPCA, sigmoid curves showing the correlation between the dose and viability were drawn. Three cell lines were seeded in a 96-well plate at a density of 2,000 cells/well, incubated for 24 h, and treated with the indicated concentrations of the drug. A cell viability assay was performed after 72 h using the Cell Counting Kit-8 (Dojindo, Kumamoto, Japan). The cell survival fraction is expressed relative to that of untreated cells, set at 100, and is shown as the mean ± standard deviation (n = 5). The 50% inhibitory concentration (IC50) was determined as described previously [26] from three independent experiments for each cell line.

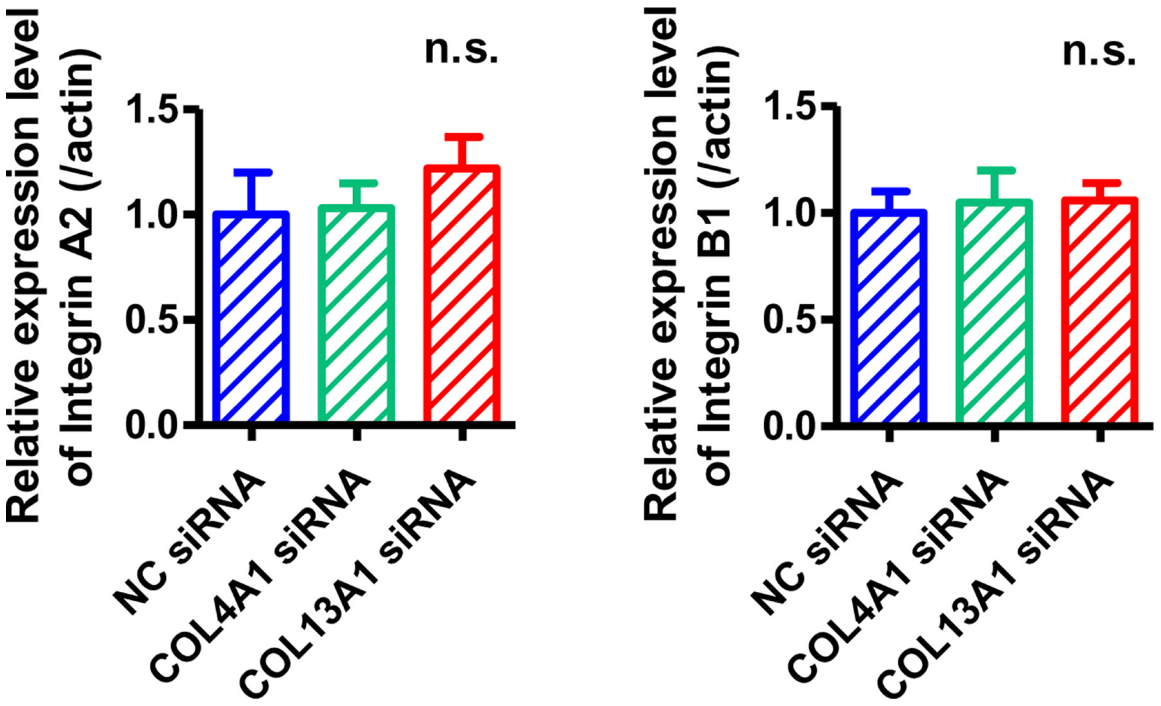

Supplementary Figure 2: mRNA expression levels of genes encoding integrins A2 and B1 after siRNA transfection. RNA extraction and SYBR green real-time RT-PCR using the comparative Ct method were performed as previously described [19]. Fold-changes in mRNA levels were determined after normalization to β-actin as the relative value vs. cells transfected with NC siRNA, which was set to 1.0. The gene-specific primer sets used in this study were: ß-actin forward 5′-CTG GAA CGG TGA AGC TGA CA and reverse 5′-CGG CCA CAT TGT GAA CTT TG, integrin A2 forward 5′-AGG TGA GAG GGA GCA GAA CA and reverse 5′-AGA CCA TTC ACA GTC CCA GG, and integrin B1 forward 5′-ATC CCA GAG GCT CCA AAG AT and reverse 5′-CCC CTG ATC TTA ATC GCA AA.

Supplementary Table 1: The assciation between COL4A1/COL13A1 overexpression and T category

T category	*n*	COL4A1		*P* value	COL13A1		*P* value
		Low	High		Low	High	
Total	97	39 (40%)	58 (60%)		45 (46%)	52 (54%)	
T1	39	19 (49%)	20 (51%)	0.045	15 (38%)	24 (62%)	0.041
T2	27	11 (41%)	16 (592%)		18 (67%)	9 (33%)	
T3	21	8 (38%)	13 (62%)		10 (47%)	11 (53%)	
T4	10	1 (10%)	9 (90%)		2 (20%)	8 (80%)	

Supplementary Table 2: Clinicopathologic characteristics of 97 cases with bladder cancer

Variables	Cases	%
Total	97	100%
Sex		
Male	77	79%
Female	20	21%
Age at dianosis		
< 60	9	9%
60 to 70	39	40%
> 70	49	51%
Median (IQR)	71 (64–76)	
T category		
T1	39	40%
T2	27	28%
T3	21	22%
T4	10	10%
Growth pattern at the invasion front		
Nodular	8	8%
Trabecular	33	34%
Infiltrative	56	58%
Concomitant CIS		
Absent	57	59%
Present	40	41%
LVI		
Ngative	72	74%
Positive	25	26%

IQR = interquartile range; CIS = Carcinoma in situ; LVI = lymphovascular invasion.

Supplementary Table 3: Antibodies used for immunohistochemical staining analysis and Western blotting analysis

Antigen	Provider	Catalogue number	Clonality	Dilution rate used in this study
COL4A1	Millipore	AB769	Goat polyclonal	1:400 (IHC), 1:100 (WB)
COL13A1	Atlas Antibodies	HPA050392	Rabbit polyclonal	1:1,000 (IHC), 1:1,000 (WB)
E-Cadherin	Cell Signaling Technology	3195	Rabbit monoclonal	1:1,000 (WB)
N-Cadherin	ZYMED	33-3900	Mouse monoclonal	1 : 500 (WB)
Vimentin	Cell Signaling Technology	5741	Rabbit monoclonal	1 : 500 (WB)
phospho-MAPK	Cell Signaling Technology	9101	Rabbit polyclonal	1 : 1,000 (WB)
total-MAPK	Cell Signaling Technology	4695	Rabbit monoclonal	1 : 1,000 (WB)
phospho-AKT	Cell Signaling Technology	4060	Rabbit monoclonal	1 : 1,000 (WB)
total-AKT	Cell Signaling Technology	4685	Rabbit monoclonal	1 : 1,000 (WB)
Dynamin	Cell Signaling Technology	2342	Rabbit polyclonal	1 : 1,000 (WB)
Actin-beta	Sigma-Aldrich	A5441	Mouse monoclonal	1 : 10,000 (WB)

IHC = immunohistochemical stainning; WB = Western blotting; MAPK = p44/42 MAPK (Erk1/2).
